# Digitalization and the third food regime

**DOI:** 10.1007/s10460-020-10161-2

**Published:** 2020-10-13

**Authors:** Louisa Prause, Sarah Hackfort, Margit Lindgren

**Affiliations:** Department of Agriculture and Food Policy, Research Group BioMaterialities, Humboldt-Universität Zu Berlin, Berlin, Germany

**Keywords:** Food regime, Digital agriculture, Agri-food system, Food commodity chain, Agrarian labor, Digital platforms

## Abstract

This article asks how the application of digital technologies is changing the organization of the agri-food system in the context of the third food regime. The academic debate on digitalization and food largely focuses on the input and farm level. Yet, based on the analysis of 280 digital services and products, we show that digital technologies are now being used along the entire food commodity chain. We argue that digital technologies in the third food regime serve on the one hand as a continuation of established information and communication technologies, thus deepening certain features of the existing food regime such as the retail sector’s control over global commodity chains. On the other hand, digital technologies also introduce new forms of control and value extraction based on the use of data and pave the way for large tech companies to take over market shares in the agri-food sector. Finally, we find that multinational agri-food companies are starting to take on the business models of leading digital tech companies, for instance by developing digital platforms throughout the agri-food system. We argue that this shows that the broader economic restructuring of neoliberal capitalism towards digital capitalism is also making its way into the agri-food system.

**Electronic supplementary material:**

The online version of this article (10.1007/s10460-020-10161-2) contains supplementary material, which is available to authorized users.

## Introduction

The digitalization of agriculture is widely hailed as the next agricultural revolution that will change how food is produced and consumed (e.g. Trendov et al. 2019). Both political and corporate leaders argue that digitalization offers the solution to feeding a growing world population, while at the same time mitigating the negative environmental and climate consequences of (industrial) agriculture (see Newell and Taylor 2017). The digitalization of food production has become a key component of various governments’ recent bioeconomy strategies and is being pushed in international fora such as the United Nations’ Food and Agriculture Organization (FAO), which is about to establish an International Digital Council for Food and Agriculture (FAO 2020b).

The academic literature offers a slightly more nuanced picture. Digital agriculture might help farmers to be more precise with inputs by offering information on ecological conditions through precise weather forecasts or sensors scanning the soil (for an overview, see Klerkx et al. 2019; Lezoche et al. 2020). Furthermore, farms will be able to reduce labor costs through the use of robotics or (semi-)autonomous machines. This will influence agrarian labor conditions and might lead to job losses in the sector (Carolan 2019). This in turn might translate into more people leaving rural communities to find jobs in urban centers (Rotz et al. 2019). Digital agriculture also raises questions of data security and sovereignty as well as farmer autonomy (Fraser 2019; Bronson and Knezevic 2016; Wolfert et al. 2017), and might enhance inequalities between farmers (Klerkx and Rose 2020).

Several studies stress the importance of looking at digitalization within the agri-food sector as a whole (e.g. Bronson and Knezevic 2016). ‘Agriculture 4.0′ is a term given to this digital transformation that indicates “potentially game-changing technologies that can dramatically affect the way food is produced, processed, traded, and consumed” (Klerkx and Rose 2020, p. 14). Nevertheless, a large proportion of the literature on the digitalization of the agri-food system still focuses on the digitalization of farming. Other parts of the agri-food system like food processing, trade, transportation or retail and consumption have received less attention (but see Carolan 2018 and Fraser 2020 on food retail and consumption; Vanderroost et al. 2017 on food packaging; Lezoche et al. 2020 on supply chains).

The food regime concept was originally developed by critical agrarian studies scholars in the late 1980s as a way to focus on the agri-food system as a whole and to identify the “historicized logic to the complexities of agro-food economics and politics” (Pritchard 2009, p. 221). Since the beginning of the 2000s, the food regime approach has received renewed interest, as global food politics is discussed in view of the characteristics of a third food regime. Contributions acknowledge the importance of information and communication technology (ICT) for the development of global food commodity chains—a key feature of the third food regime. However, these debates have so far not fully engaged with the recent development of new technologies, particularly digitalization. McMichael (2019) mentions the increasing importance of “bio-capitalism (…) and the technologization of nature and digitalization” (133), Dörr (2018) notes corporate concentrations in the area of precision farming, and Newell and Taylor (2017) present an interesting discourse analysis on ‘climate-smart’ agriculture. Yet a more systematic engagement with digital technologies is so far missing.

In this article, we bring together the debates on digital agriculture and the third food regime in order to provide a better understanding of how digital technologies are changing the agri-food system. This is particularly relevant since recent contributions on the third food regime (e.g. McMichael 2019; Tilzey 2019) point to the transition of some features of the regime, to which the rise of digital technologies might contribute. Our central research question is how digital technologies shape the organization of the third food regime. Our aim is first to provide a broader picture of the digital technologies that are currently applied in food commodity chains, and secondly to evaluate where digital technologies act as a mere ‘update’ of ICTs, and if and where we can identify potential shifts in the current organization of the agri-food system due to digital technologies. We answer our research question through an empirical analysis of 280 key digital technological products and services currently offered in one or several steps of the global food commodity chain. We focused our analysis on food crops, and excluded dairy production and processing as well as livestock raising, meat production and aquaculture. We also excluded developments in urban agriculture from our data set, since we believe that to date their contribution to the global production of food remains limited.

The article is structured as follows. First, we outline our theoretical framework, which is based on debates around the third food regime. Second, we provide an overview of our data selection and analysis. In the empirical part of the paper, we identify key digital technologies for each step of the food commodity chain and highlight how they are changing the organization of the current food regime. Finally, we conclude by reflecting on continuities and changes in the third food regime, and highlight the importance of digitalization as both a continuation of ICTs and in terms of facilitating a transition towards new forms of generating value and corporate control.

## Theoretical framework

In order to better understand how digitalization might change the current agri-food system, we build upon the food regime concept as developed chiefly by Friedmann and McMichael (1989). Food regimes, though relatively stable, are also contingent, contested and temporary “constellations of governments, corporations, collective organisations, and individuals that allow for renewed accumulation of capital based on shared definition of social purpose by key actors, while marginalising others …” (Friedmann 2005, p. 228). How a food regime is organized has important implications for farmers, food workers and consumers, and shapes the power relations between these groups and agri-food corporations (Friedmann 2005, p. 228).

The analysis of food regimes is methodologically consistent with commodity chain approaches in the tradition of Friedland (1984), since it traces production relations globally. Yet it also goes further in terms of integrating consumption and state actors—issues that have long been neglected in commodity chain approaches (Dixon 1999; Friedland 2001). Food regime analysis offers an analytical lens to grasp the broader economic and (geo-)political relations, the discursive legitimations, rules, norms and regulations, social forces such as social movements, and the technical and environmental changes that organize food production, consumption and distribution in historical constellations of power and accumulation (Magnan 2012; Bernstein 2015). Thus, rather than tracing the relations of production for a specific commodity, it enables us to comprehensively analyze the organization of the agri-food system at the macro-level. While this approach admittedly omits local-level developments, it is nevertheless useful for our analysis since it offers an analytical lens to situate digital technologies as new forces shaping the production, distribution and consumption of food in the socio-economic and political context of the current agri-food system in a historically-informed and comprehensive way.

The approach distinguishes three different global food regimes that have been interrupted by periods of transformation and instability: the colonial food regime, which corresponded with British imperial rule from the 1870s to 1914; the second food regime of the post-war period from 1947 until 1973, which corresponded with American imperialism; and the third food regime, which has been identified for the period from the creation of the WTO in 1995 until today, and has been dubbed the ‘corporate food regime’ by McMichael (2009) or the ‘corporate-environmental food regime’ by Friedmann (2005). McMichael himself, however, states that the “corporate food regime is in transition” (2019, p. 118). Many of the dominant relations of the third food regime are still in place, while new ones are emerging (McMichael 2019, p. 133). Digital technologies are thus entering the agri-food system in a period where its organization is unstable and contested, and they might play an important role in current reconfigurations of the food regime. Both the first and second regimes have been described extensively elsewhere (e.g. Friedmann and McMichael 1989; Friedmann 2005; McMichael 2009). We therefore focus on identifying the key characteristics of the third food regime that are still in place, and point to both historical continuities as well as new emerging relations.

The third food regime is characterized by neoliberal, free market policies as the dominant mode of regulation and is driven by global market prices (McMichael 2009). However, the ongoing blockade of WTO negotiations, the recent collapse of its Appellate Body, as well as the return of “agro-security mercantilism” (McMichael 2012) in the form of land grabbing all show that neoliberal hegemony is fragmenting (Tilzey 2019). Nevertheless, WTO rules still apply and constitute a residual feature of the third food regime, while emerging developments include bi- and multilateral trade agreements as well as various corporate and financial deals and mergers with and across states (McMichael 2019). Unlike the two previous regimes, the third does not feature one hegemonic nation state, but points to multipolarity, with China being the most important new player on the agri-food stage alongside Brazil and India (McMichael 2019).

The third food regime is characterized by the rapid expansion of global food commodity chains. Many countries of the Global South are now incorporated into commodity chains as sources of cheap processed foodstuffs on the one hand and of fresh fruit and vegetables for an affluent consumer class in the Global North on the other (Burch and Lawrence 2009, p. 275). This has resulted in more flexible systems of production, often based on contracts, extensive shift work, and flexible and precarious working conditions for farm workers, with the aim of achieving continuous production and stabilizing constant and reliable supply to the major retailers. Global food commodity chains are controlled by large supermarkets, which have greatly increased their power compared to their position in the second food regime and have taken over certain regulatory functions from state actors, including food quality and safety standards (Friedmann 2005). In response to a growing number of increasingly environmentally-conscious and affluent consumers in the Global North, corporate actors, particularly supermarkets, are legitimizing their actions through narratives of environmental sustainability, which exist side-by-side with narratives developed during the second food regime that stress modernization, food security and increased yields (Bernstein 2015). In order to legitimize their products as environmentally friendly, a number of supermarket-driven private certification schemes have been developed, which have taken over regulatory functions from the state.

Large agri-food companies formed during the second food regime now dominate the production of farm inputs, food trade, processing and retail, and there is even greater corporate concentration through mergers and takeovers (Dörr 2018). Closely linked to the feature of corporate power is the issue of financialization and the increasing influence of finance capital on the agri-food system (Burch and Lawrence 2009). Established agri-food companies, start-ups as well as land itself have become the targets of speculative investments by asset managers (McMichael 2019).

The third food regime inherited from the second regime the dominant production model of fossil-fuel-driven, large-scale and capital-intensive agro-industrial farms. This model was exported to the Global South as the ‘Green Revolution’ (Patel 2013), and since the mid-2000s has been expanded through another round of enclosures, also referred to as the ‘global land grab’ (Prause 2020). The expansion of industrial agriculture and global food trade in the third food regime is contributing significantly to the advancement of the climate crisis and the marginalization of the cultural and ecological knowledge of small-scale farmers (McMichael 2009). The corresponding tension between an agro-ecological small-scale model of farming and large-scale industrial agriculture is crucial. Food sovereignty and agro-ecology movements around the globe are struggling to enact a different mode of agricultural production and a reorganization and democratization of the agri-food system towards more localized, ecological means of food production and consumption, which would guarantee the sovereignty and well-being of family farmers and sustain nature’s ability to reproduce itself (Friedrich et al. 2019; Prause and Le Billon 2020).

The rise of global commodity chains in the third food regime was enabled by the interplay of trade liberalization and the establishment of a new ICT infrastructure during the dot-com boom of the 1990s, which made possible the coordination and exchange of information within complex global networks. Technological developments in cooling, preserving and transportation were likewise preconditions for guaranteeing year-round access to fresh produce for affluent consumers (McMichael 2009). Mechanization, advances in plant research, the use of chemical inputs and seed modification were the driving technologies of the second food regime, and continue to be so in the third. Further key technologies in the third food regime include advances in biotechnologies, particularly the development of genetically-modified seeds, which have further increased corporate power (Pechlaner and Otero 2008).

New technologies therefore contribute to shifting power relations between food producers, consumers, states and corporate actors, and in conjunction with rules and regulations play an important role in the organization of food regimes. What is missing in the literature so far is discussion of a new set of technologies—digital technologies—that are making their way rapidly into the agri-food system in a period where the third food regime is in transition. To structure our analysis of digital technologies, we distinguished the following steps within a food commodity chain (following Lang and Wiggins 1985): the production of agricultural inputs, including fertilizers, pesticides and plant breeding, including GMO seeds; the farming process, including irrigation and crop growing; the trade of raw agricultural produce; food processing, where raw agricultural products are manufactured into finished food products, as a basic or highly processed commodity; food packaging; transport and storage; and finally food retail and consumption, which includes major supermarkets and smaller independent traders selling produce directly to end consumers.

## Methodology

Academic literature has largely neglected the digitalization of the agri-food system beyond the farming sector, and peer-reviewed articles often only provide a few examples of concrete digital technologies. In order to gain an overview of important technological developments, we based our data corpus on reports mainly compiled by non-academic institutions and on an extensive online search of the websites of agri-food companies that sell digital technologies. We gathered our data between February and April 2020, using Google, Google Scholar and Scopus as our prime search engines.^1^ Our aim was to include reports by a wide diversity of actors to gain a comprehensive overview of the digital technologies applied in the agri-food system. We therefore included studies issued by corporate actors, NGOs, government agencies, consultancies and multilateral organizations.^2^ We also included in our database technologies that were mentioned in academic peer-reviewed articles; this was particularly relevant for those steps in the food commodity chain where we found only a few reports published by non-academic actors (e.g. food packaging).^3^

We found 92 studies in total, of which we identified 53 as relevant for our analysis, all published between 2011 and 2020. Our selection criteria were based on relevance to our research question, the report’s quality, and the description of digital technologies already on the market. Based on our reports, we identified and categorized 280 services or products based on digital technologies provided by 197 companies, start-ups and public and governmental institutions that are being applied across the entire food commodity chain. We do not claim to provide a comprehensive overview of all digital technology products that are currently used in the agri-food system. Rather, our aim is to give an overview of some of the key technologies that are currently being applied and to discuss their impacts for the third food regime. For our analysis, we first sorted the digital products according to their place in the food commodity chain. We then researched the product and the company offering it online. Based on this search, we categorized the companies according to their sector and size, and researched the main investors where applicable. We also added additional information on the types of agriculture that these technologies were developed for and the narratives used by corporate actors. Finally, we added additional information on the purpose and function of the products and services to build clusters that grouped together a range of products based on digital technologies that offer a similar kind of service to the farmer/consumer/food processor etc., even though they might differ in their respective functions.

## Digital technologies along the food commodity chain

Our empirical research shows that digital technologies are now used along every step of the food commodity chain. Table 1 summarizes the most important clusters of digital products and services that we found.

**Table 1:** Digital technologies along the food commodity chain

Step in food commodity chain	Key digital product or service	Key actors and example companies
Agricultural inputs	Fintech for credit evaluation and payment services	Start-ups (e.g. Advans Group); non-profit start-ups (e.g. One Acre Fund)
	Data-based insurances	Agriculture insurance companies (e.g. AIG Crop Risk Services)
	Genome-edited seeds	Start-ups (e.g. Calyxt); agro-chemical corporations (e.g. DowDuPont)
Farm operations	Precision agriculture equipment	Start-ups (e.g. Blue River Technology); agro-machine and equipment companies (e.g. John Deere); agro-chemical companies (e.g. Yara International)
	Farm robotics	Start-ups (e.g. Naio Technologies)
	Digital machine-sharing platforms	Start-ups (e.g. Tro Tro Tractor); agro-machine and equipment companies (e.g. Tractors and Farm Equipment Limited)
	Data-based agronomy advice and information	Start-ups (e.g. Indigo Ag); social start-ups (e.g. Green Dreams Tech); agro-chemical companies (e.g. Bayer Crop Science); public institutions (e.g. FAO)
	Farm management platforms	Agro-chemical companies (e.g. Syngenta); agro-machine and equipment companies (e.g. John Deere); start-ups (e.g. CropX)
Primary commodity trade	Digital marketplaces	Start-ups (e.g. Indigo Ag); multinational tech companies (e.g. Alibaba); multinational food trading corporations (e.g. Cargill)
Food processing	Collaborative robotics	Food processing companies (e.g. Nestlé)
	3D food printing	Food processing companies (e.g. Choc Edge)
Packaging	Smart packaging	Tech companies (e.g. Adobe Inc)
	3D printing for polymer-based materials	Tech companies (e.g. MakerBot Industries, LLC)
Transport	Quality sensors and analytics	Logistics companies (e.g. Purfresh); tech companies (e.g. Tellspec)
	Digital freight management	Multinational food trading companies (e.g. Cargill)
	Digital transport logistics for small-scale producers	Farmer organizations (e.g. Zambia National Farmers' Union); start-ups (e.g. Distrego)
Storage	Automated warehouses	Supermarkets (e.g. Ocado); food processing companies (e.g. Nestlé)
Retail and consumption	Smart shopping	Supermarkets (e.g. Carrefour); tech companies (e.g. Amazon)
	E-commerce platforms	Tech companies (e.g. Alibaba); supermarkets (e.g. Wholefoods Market)
Entire commodity chain	Digital tools for commodity chain traceability and transparency	Supermarkets (e.g. Carrefour); tech companies (e.g. Amazon); farmer organizations (e.g. Ugandan National Union of Coffee Agribusiness and Farm Enterprises); food processors (e.g. Nestlé); food commodity traders (e.g. Louis Dreyfus)

The two most significant digital developments taking place at the input level are genome-edited seeds, using for example Crispr/Cas9, and the use of innovative financial technologies (fintech). While most of these genome-edited seeds have not yet been commercialized, large agro-chemical companies are currently racing to secure the patents (Then 2019: 11). Fintech companies rely on a wide range of social and environmental data to determine smallholder farmers’ creditworthiness and to administer insurance services. At the farm level, precision-agriculture equipment, robotics, agronomy advice and information—such as weather apps or weed identification apps—and farm management platforms make up the main digital developments. These products are often sold with the promise of offering a more precise use of inputs, reducing the required labor and producing higher yields, and are based on the use of big data analysis and Internet of Things (IoT) technologies, as well as artificial intelligence (AI) (see also Klerkx and Rose 2020; Lezoche et al. 2020). Farm management platforms in particular have attracted investment from large agri-food companies.

Digitalization is also taking place in food commodity trading, processing and storage. A range of digital marketplace platforms, mostly developed by start-ups, are offering to bring farmers and buyers, or input suppliers and farmers, together. These marketplaces aim to minimize the role of middlemen, and/or differentiate the commodity market as a source of new value creation (Mitchell 2019). At the food processing level, the main trend in emerging digital technologies involves automation or robotics, such as optical systems that automatically sort fruits and vegetables, and IoT technologies such as collaborative robots that communicate with one another (Nestlé 2019). Some companies are also experimenting with smart packaging, computer-aided automation and 3D printing (Vanderroost et al. 2017). In food transportation, different types of quality sensors and analytics allow for greater control over the condition of the food being transported or stored in order to decrease food safety risks, increase transparency and avoid losses through spoiling. In the food storage sector, we observed the emergence of automated warehouses (see also Fraser 2019).

Further downstream in the commodity chain, our data analysis showed three main digital trends for the retail and consumption sector: the use of data to enhance the traceability and transparency of the food commodity chain; smart shopping; and a wide variety of e-commerce platforms offering groceries online. Several large retailers (e.g. Walmart, Carrefour) are experimenting with digital technologies to trace the supply chains of specific products and make them transparent to the consumer via digital ‘passports,’ or through blockchain technologies and QR codes. Some of these technologies also allow retailers to collect consumer data and thus to forecast product-ordering levels and generate individually-customized offers or individual pricing, thereby influencing consumer behavior (see also Carolan 2018). We also observed large multinational tech companies like Amazon and Alibaba moving into online food retail and offering grocery delivery options.

Our research suggests that the digital transformation of the food system is largely driven by multinationals from the agri-food and tech sector, as well as private sector start-ups. The digital technologies developed for this transition differ according to accessibility, the required hardware and infrastructure, the associated costs and the involved actors. One common trend in the range of technologies throughout the commodity chain is the growing dependence on extracting and analyzing large amounts of data. In the following sections, we analyze how the different technologies we identified, as well as the increased dependency on data, shape key features of the third food regime.

### ‘Supermarketization,’ green narratives and tech companies

Supermarkets have become key actors in the control of global food commodity chains in the third food regime. They have taken over regulatory and norm-setting functions regarding standards for high-quality and high-value foods such as fresh produce, and more recently also for low-value foods such as corn and soy, that were previously performed by national governments (Burch and Lawrence 2009; Freidberg 2020). Digitalization does not alter the existing basic principles of traceability and quality standards, but it is advancing them through new tools that allow for more precise product tracing. For instance, food commodities are often sold in bulk, making them hard to trace beyond their wider region of origin, unless the retailer is directly involved in production. Digital supply chains make it possible to record and store precise information regarding the harvest date for a specific fruit, the location and owner of the plot, when it was packed and how long it took to transport to Europe (Thomasson 2019). Such technologies might provide some degree of transparency for end consumers; however, they also allow for increased data and information extraction by corporations and might have exclusionary effects for some farmers. If a farmer’s products are considered sub-standard, for instance, they can be targeted as individuals rather than as part of a cooperative or larger farming community. Cargill, one of the largest international food traders, uses GPS mapping to obtain detailed information about farm location, size, cultivation methods, as well as farmers’ choices about fertilizers and replanting activities, “along with a wealth of information about farming families and communities” (Cargill n.d.). Such information allows food retailers to market their commodities to consumers as being both ‘safe’ and ‘green,’ and represents a continuation of the corporate-environmental repositioning that began in the second food regime.

In the context of digitalization, the environmental narrative also serves a second function, namely to legitimize the introduction of digital technologies along the global commodity chain. Cargill writes on its website that it uses GPS mapping for more than 56,000 smallholder farms in Côte d’Ivoire, Indonesia and Cameroon “to demonstrate whether a farm location is linked to a deforestation hotspot” as part of their “commitment to eliminating deforestation from [their] cocoa supply chain” (n.d.). The start-up Farmforce claims that the purpose of its blockchain technology for commodity chain traceability “is to deliver digital solutions to secure sustainable sourcing, […] and protect the environment” (n.d.). Farm machinery equipment companies and input producers claim that digitalization is making their products ‘climate smart’ (Newell and Taylor 2017). Thus, environmental narratives are legitimizing a digital transition in the food system that might otherwise raise critical questions about issues such as data sovereignty, increased surveillance and corporate control over farming practices.

Linking ‘sustainability’ to the application of digital technologies also allows corporate actors to gain institutional support for their technological developments and to consolidate and advance their control over technologies, livelihoods and food production, while at the same time marginalizing agro-ecological alternatives in international fora and institutions (Newell and Taylor 2017). Agri-food corporations have successfully pushed a ‘climate-smart narrative’ into UN institutions such as the FAO. This is mirrored in the recent announcement by member states of the Global Forum for Food and Agriculture that they will set up an International Digital Council for Food and Agriculture under the auspices of the FAO. One rationale put forward is the aim to “create a more efficient and equitable global agri-food system that would help in achieving the Sustainable Development Goals (SDGs)” (FAO 2020b, p. 2). The EU has also adapted this approach to digital agriculture, stating in a declaration that “Digital technologies (…) have the potential to increase farm efficiency while improving economic and environmental sustainability” (European Commission 2019). At the same time, the EU announced its financial support for the sector through funding for research and development and the establishment of “a Europe-wide innovation infrastructure for a smart European agri-food sector and a European dataspace for smart agri-food applications” (FAO 2020b, p. 2).

Digital supply chains seem to stabilize rather than challenge retailers’ control over global commodity chains. At the same time, we observe a shift within the retail sector, largely facilitated by digital technologies, that might threaten the market dominance of traditional supermarkets and contribute to the transitional forces within the third food regime. Capital from the tech sector is increasingly invested in the retail sector and is starting to take over market shares. Until recently, large supermarket chains relied on established oligopolies in many countries and very little competition. In the US in 2015, the four largest retailers accounted for about 40% of national grocery sales, while in the European Union in 2011, the top-five retailers in 13 member states accounted for about 60% (IPES-Food 2017, p. 45). With regard to the investors, the concentration is even higher, with only five big asset managers (among them BlackRock and Vanguard) owning a large portion of company shares in the retail sector (ETC Group 2019). Spurred by the opportunities of digitalization, several large tech companies have moved into the retail sector to provide e-commerce services for groceries, thereby weakening supermarket chains’ market dominance. In addition to the world’s top grocery retailers such as Walmart and Tesco, Amazon and Alibaba have also bought into food grocery e-retail (Kumar 2018). With its purchase of Whole Foods Market and its use of big data to track consumer behavior and preferences, Amazon might become one of the world’s top-10 food retailers (IPES-Food 2017, p. 45). We thus believe that we are currently witnessing a shift in corporate power away from the supermarkets towards actors in the tech sector, facilitated in part by digital technologies.

Tech companies are not only active in the retail sector. The Chinese company Alibaba, for example, also offers the ET Agricultural Brain, an AI-based tool for enhancing fruit and vegetable planting, while the Alibaba Blockchain Food Trust Framework offers blockchain technologies for food traceability and transparency. We also found digital technologies for data analysis, AI and machine learning developed for agriculture by Microsoft, IBM and SAP. While they do not yet seem to be threatening the traditional agro-chemical and farm machinery companies, tech companies are also playing an increasingly important role at the downstream end of the commodity chain. At the forefront of this movement of tech companies into the agri-food sector via digital technologies seem to be US and Chinese companies, with SAP being a European exception.

### Financialization

Several definitions of the third food regime see not only supermarketization but also financialization as its defining feature (e.g. McMichael 2009; Burch and Lawrence 2009). These authors argue that the third food regime reflects the overall characteristics of neoliberal capitalism, in that financial institutions and instruments are increasingly involved along the agri-food commodity chain. We believe this is also true in the area of digitalization. Not only have investment companies been instrumental in some of the biggest mergers in the agri-food system, such as the acquisition of Whole Foods Market by Amazon, but financial investment companies also own a growing number of shares in large agri-food companies that control key digital technologies in the food commodity chain, such as farm management platforms (Dörr 2018).

Since the 2008 financial crisis, the agri-food sector has become an attractive and relatively secure investment option for financial capital (Burch and Lawrence 2009). In 2019, for example, BlackRock held shares in many major agri-food businesses, amongst them 7.2 percent of voting rights for Bayer-Monsanto and 6.3 percent ownership of Corteva Agriscience, a subsidiary of DowDuPont (Jessop and Burger 2019), therefore wielding increasing market power in the sector (ETC Group 2019). Financial investors have also been a driving force behind the establishment of many of the start-ups offering digital technologies. In our analysis, we found 57 start-ups offering digital technologies for one or several steps of the food commodity chain. Many of these start-ups lack a clear profitability model, relying instead on investment money in their attempt to capture market shares (PA Consulting 2018). The start-up Indigo Agriculture, for example, had raised a total of $850 million by the end of January 2020 from corporate investors such as FedEx and Activant Capital, as well as public investment funds such as Investment Corporation of Dubai and Alaska Permanent Fund (Somerville 2017; Indigo Agriculture 2020, 2017).

Traditional agri-food companies are also building up capital venture arms that invest directly in digital agricultural start-ups. Many of the established multinational companies in the food system, such as Syngenta, Bayer, John Deere and Cargill, keep track of promising new digital innovations by collaborating with, or establishing their own, start-up incubators and accelerators, which connect start-ups or university-based research groups with venture capital firms and corporations, to assist in bringing technological innovations to the market and allowing the agri-food multinationals to invest in or absorb promising technologies at an early stage. For example, Blue River Technologies, a company that uses AI to automatically identify and spray herbicide on weeds, was initially funded by Syngenta’s venture capital arm and later bought by John Deere in 2017. As such, it is not simply that financial investors are expanding into the agri-food sector, but that established companies in the sector are themselves increasingly adopting the logic of finance capital (see also Burch and Lawrence 2009 for the retail sector).

A very different aspect of financialization touches upon smallholder agriculture and the involvement of new actors in evaluating creditworthiness. We found a range of fintech start-ups that base their services on the extraction and analysis of data. The start-ups Farmdrive and Advans both use digital technologies to assess farmers’ creditworthiness based on psychometric data as well as farm operations and environmental data. Both companies claim that this digitalization of financial services will help historically disenfranchised smallholder farmers to access financial services. Rather than being solutions for ‘financial inclusivity’ for disenfranchised populations, however, research suggests that these new systems of credit scoring have stratifying and disciplinary tendencies (Fourcade and Healy 2017; Roderick 2014), and can contribute to different neoliberal or authoritarian forms of ‘algorithmic governance’ (Gruin 2019). They also rely on already established financial infrastructures that continue to reproduce, or even deepen, global inequalities (Bernards 2019). As such, these data-driven financial technologies perpetuate existing systems of control.

### Corporate power and the ‘data grab’

Our data shows that large agri-food and tech corporations are by now controlling many of the key digital technologies along the food commodity chain. At the input level, we observe the traditional big players of the seed markets, Bayer, DowDuPont and Syngenta, taking control of new genome editing technologies. DowDuPont holds the highest number of patents on these new technologies and is thus able to offer bundled, non-exclusive licenses to a patent pool—resulting in considerable market control and power. DowDuPont currently also leads in international patent applications (filed with the World Intellectual Property Organization) in the field of genome editing; Bayer follows in second place before Calyxt, which is marketing the first soybean modified using new genetic engineering techniques. Also included are Syngenta and BASF, while a few patents have also been filed by traditional breeding companies such as KWS (Then 2019; Cameron 2017).

Beyond such ‘traditional’ forms of corporate control through intellectual property rights, we believe that digitalization also allows for new forms of corporate control through the collection and privatization of big data. Big data refers to large flows and stores of data that are generated continuously with the aim of being exhaustive and fine-grained in scope, and flexible and scalable in production (Kitchin 2014, p. 2). A central node for the collection of data are farm management platforms that collect data points from individual farms. In order to gain access to the benefits of the technology, farmers have to reveal their agricultural knowledge about soil fertility and crops, as well as personal farm details. Monsanto’s FieldScript program, for example, requires two years of farm data on yields, soil quality and field mapping before the farmer can access any beneficial services (Schimpf 2020). Farm management platforms often offer multi-tiered service packages, sometimes with a free basic version designed to attract a critical number of users to capture market shares. Farm management platforms, like other digital platforms, create lock-in effects for their users, so that while the costs of using the platform are low or nil, the costs of switching to a different provider are high (for example, due to the incompatibility of data formats).

In using most of the platforms we analyzed, individual farmers hand over control of their data to the company. If they can access their own data at all (many platforms do not disclose their back-end processes and data to customers, including information about how customers’ data is used and for what purposes), they often lack the tools and capacities to analyze it. It is thus corporations that are benefiting from big data collection and analysis (Carbonell 2016), leading Fraser (2019) to talk about the privatization of data and a ‘data grab’ in digital agriculture. This is made possible in part through a lack of regulation. In the context of digitalization, the law around data and privacy becomes an important aspect of agricultural regulation. Recent attempts by the US Farm Bureau to ensure the security, ownership and protection of farmers’ data when they use farm management platforms have been undermined by corporations. Bayer, for example, adjusted its End User License Agreement for its platform to ensure that it retains control of farmers’ data for further use (Schimpf 2020). The absence of stricter rules currently adds to data-based corporate control in the agri-food system.

The privatization of data through farm management platforms offers companies several possibilities to create value. Input providers such as Syngenta are linking their traditional products, like seeds and pesticides, ever more closely to the farm management services they offer. Syngenta has invested heavily in the takeovers of farm management platforms in the past five years, and claims to be the only company with access to the leading farm management platforms in the world’s top four agriculture markets: the US, Brazil, China and Eastern Europe. Syngenta estimates that around 28 million hectares of farmland are managed by its platforms. However, Syngenta’s chief information and digital officer explained in an interview that Syngenta does not believe that it can make money from selling the software to farmers; instead, the company sees farm management platforms as accompanying its core products in crop protection and seeds (Rana 2020). Thus, Syngenta is using the data it collects to optimize its products and gain an advantage over its competitors. Simultaneously, it is creating further lock-in effects for farmers, such as when Syngenta inputs no longer work without the platform and vice-versa, or when platforms provide strong incentives to grow a particular crop to which the technology is best adapted (see also Carolan 2020).

The collection of data through farm management platforms also offers the opportunity to compile a large aggregated data set on farming that might be sold to different farm input suppliers, agronomists and machinery firms, but also to national and international agricultural political institutions. Start-ups like SatSure or MyCrop offer policy consultancy based on the farm data they collect. Thus, the ‘data grab’ might not just influence company-farmer relations, but could also have an influence on policy-making.

Our data shows that all of the large agro-chemical and agro-machinery companies have taken over or developed at least one farm management platform. Next to Syngenta and its four platforms, Bayer is an important player through its ownership of Climate Field View, which in 2018 had more than 100,000 registered clients in the US, Canada and Brazil, who together farm about 120 million acres (Carbonell 2016; McDonnell 2014). Climate Field View is compatible with the farm management tools offered by John Deere and AGCO. CLAAS as well as BASF and Corteva Agriscience also own such platforms. This indicates the importance that the large companies in the sector attribute to this digital product, and how, according to Monsanto’s chief technology officer Robb Fraley, “the information itself becomes the big business” in agriculture (McDonnell 2014).

Corporate control over the agri-food system is thus increasingly tied up with control over big data, which offers agri-food companies the opportunity to use data to enhance their own products, to bind farmers more closely to the companies’ products, and to extract value from selling aggregated data and possibly gaining political influence.

### Labor and production

Farm management platforms also have the potential to (re-)shape the relationship between farm owners and farm workers. Digital technologies can foster a labor model, dubbed ‘digital Taylorism’ or ‘Neo-Taylorism,’ that comprises new modes of workplace surveillance, control and worker deskilling, as well as of measurement, standardization and quantification of work (Altenried 2020). This dynamic has so far only been identified for other economic sectors, however we believe that farm management platforms offer the necessary tools to introduce digital Taylorism to the farm. The John Deere Operation Center, for example, offers a detailed machine location history, semi-standardized communication with operators and detailed ‘performance’ analysis, including live performance tracking of different fields, which allow for the comprehensive surveillance of farm machinery operators as well as a further standardization of their tasks. Furthermore, farm management platforms offer several services that might lead to a deskilling of both farm workers and farmers themselves, such as decision-support systems and agronomic advice (see also Carolan 2020), as well as guidance and steering systems that automate many of the processes and decisions that farm machinery operators previously made autonomously. We found several start-ups offering agronomic advice, such as pest diagnosis and phenotyping, targeted at small-scale farmers via low-tech ICT such as mobile phones and simple apps. This could lead to a further marginalization of the cultural and ecological knowledge of small-scale farmers, when their knowledge is replaced by data analytics and/or AI. However, while our data shows that farm management platforms and other data-based agronomic services could possibly alter agricultural labor and knowledge, whether this is the case, and at what scale, requires further empirical investigation.

Debates on the third food regime tend to focus solely on farm labor. What our analysis shows, however, is that digitalization is also very likely to change labor in other sections of the commodity chain. Amazon’s ambitions, for example, to establish cashier-free stores may change the labor market in the retail sector, whereby jobs are lost and supermarket labor restructured, possibly exacerbating intersectional inequalities by disproportionately affecting low-skilled workers and women. Automated, networked and roboticized warehouses like those of Ocado, combined with increased e-commerce and grocery delivery, seem to suggest that labor in food storage and logistics facilities might be taking on characteristics of the digital Taylorist working conditions that are already established in Amazon warehouses.

Regarding agricultural production, we believe that digitization will deepen existing tendencies towards an increased gap in terms of profitability between small- and large-scale agriculture. From the 137 products that we found for the input and farm levels, most were developed for large-scale industrial farming, such as guidance systems, (semi-)autonomous tractors and harvest robots. The latter are intended to decrease the need for farm labor even further, while increasing the productivity of large-scale farms (e.g. Harvest Croo Robotics 2020).

Our findings also indicate that the use of digital technologies at the farm level might be driven by a partial re-regulation of agriculture and food production that we are currently witnessing as part of the transitional tendencies of the third food regime. China, for example, amended its food safety laws in 2015 and established a new food safety administration (Kuhlmann et al 2019). Germany has passed new regulations regarding the use of fertilizers in order to comply with the European Commission’s requirements on groundwater quality. In 2019, the European Court of Justice classified new genome editing techniques as conventional genetic engineering, in order to ensure food safety and the protection of human health and the environment, in line with the precautionary principle (Andersen and Schreiber 2020). Furthermore, social movements have in the past years strategically mobilized against bi- or multilateral free trade agreements, which in light of the malaise of the WTO have become an even more important feature of international trade, in order to demand the integration of social, environmental and safety standards, particularly with regard to food imports. Such demands have, for example, been partially incorporated into the recent proposal for the EU-Mercosur trade agreement (Ghiotto and Echaide 2019).

In this context of an attempted (if still partial) re-regulation of the farming sector and aspects of international food trade, digital technologies, particularly farm management platforms and digital supply chain technologies, might become an important tool for farmers to generate proof that they have complied with regulatory frameworks. Such features are already provided by a number of digital service providers, for example the platform 365Farm.net by CLAAS, and organizations such as the OECD are pushing for the digitalization of border agencies and certification mechanisms to facilitate international food trade (Jouanjean 2019). This might result in the further exclusion of small-scale producers from certain markets, since it could be difficult for smaller producers to pay for certification or access the required technological tools.

Nevertheless, digital technologies are not necessarily detrimental for small-scale agriculture. Some companies are developing digital products tailored to the needs of agro-ecological farmers. In East Africa, the start-up WeFarm claims to have set up the largest farmer-to-farmer digital network, with more than 1 million users in Kenya and Uganda. WeFarm allows farmers to share questions, information and advice, and might thus strengthen local agricultural knowledge rather than marginalize it. The German-based company Rucola Soft offers a planning tool for vegetable cultivation customized to the needs of community supported agriculture. The freeware and open source solution FarmOS is designed for and can benefit smallholders. Several other similar solutions, such as AgXChange, IsoBlue, FarmLogs, the OpenAg Data Alliance and the Open Food Network, enable farmers to stay independent of large corporations and to regain or maintain data sovereignty in deciding how their data is shared (Carbonell 2016; Fraser 2019). Moreover, digital infrastructures have the potential to facilitate trans-local food movements and the sharing of place-based knowledge for the benefit of farmers and food sovereignty groups (Santo and Moragues-Faus 2019).

While digital technologies that are developed for and ideally with the help of small-scale farmers might bring some improvements for family farmers, our analysis suggests that digitalization does not signal a general departure from the large-scale industrialized and fossil fuel-dependent agricultural model characteristic of the third food regime. Even though agriculture is responsible for 20% of global greenhouse gas emissions (FAO 2020a) and has become a focal point of international political regulation around the climate crisis, it is unlikely that digitalization will bring about a more sustainable model of agriculture.

## Conclusion

Our analysis has shown that the digitalization of food production is a phenomenon along the entire commodity chain. To understand the impacts of digitalization on the organization of the agri-food system, we believe it is crucial to overcome the current debate’s tendency to focus on digitalization at the input and farm level. One reason why such a broader approach is largely missing might be that digitalization along the food commodity chain seems to be discussed under different terms and in different strands of the literature. Genome editing is generally discussed as biotechnology; automation, robotics, IoT, AI and digitalization in the food processing and packaging sector are referred to as industry 4.0; while similar technologies at the farm level are referred to as smart farming or agriculture 4.0. Bringing these three strands of the literature together might be a first step in furthering inquiries into the future of food production.

Using the third food regime as our analytical lens shows that claims about a new revolution in agriculture and food production are exaggerated. If we look at the organizing principles of the third food regime, we can see that many have been kept in place. *Supermarkets* are intensifying their control over food producers and commodity chains, for instance via digital supply chain technologies. *Green narratives* are still used by retailers to market their products, and they are now supported through, and used to legitimize, digital technologies, by stressing their alleged contribution to environmental sustainability across the commodity chain, not least as a selling point to attract consumers. This focus on environmental sustainability constitutes a residual feature of the third food regime, yet it is increasingly combined with a focus on digital technologies in the notion of ‘climate-smart agriculture.’ *Financialization* has been discussed as another key trait of the third food regime. Financial investors are driving the development of many digital technologies through large investments in digital agriculture start-ups. Furthermore, financial capital facilitates vertical integration and mergers, and the takeover by large agri-food and tech companies of smaller companies offering digital technologies such as farm management platforms. Finally, agri-food companies are increasingly adopting the logic of finance capital to establish venture capital arms of their own to invest in promising start-ups. We therefore found that the close ties between financialization and corporate control over food production also hold true for the development and use of new digital technologies. As our analysis shows, big tech and major agri-food companies dominate the technologies along the entire food commodity chain, from intellectual property rights for a new generation of GMO seeds, through farm management platforms, to the establishment of automated warehouses and consumption solutions. Thus, there is very little to indicate that digitalization will bring about profound changes in the dominant model of food production or the functioning and distribution of profits along global commodity chains. Since many of the digital technologies we analyzed were capital-intensive and often targeted at large-scale agriculture, we believe furthermore that the opposition between small-scale agro-ecological farming and large-scale industrial farming will be fortified by digitalization.

Our analysis does, however, show that digital technologies are contributing to certain transitions of the third food regime. At the *retail* end, we see tech companies increasingly moving into the sector based on digital technologies such as e-commerce platforms and GPS for delivery logistics. This is transforming the retail market, as e-commerce provides an important alternative to traditional supermarkets – a trend that might be reinforced by the Covid-19 pandemic. Digitalization seems to facilitate the stronger participation of tech companies in the agri-food system in general. Many of the large tech firms such as Amazon, Microsoft and Alibaba are now offering products at other stages in the food commodity chain. Our data shows that Chinese tech companies in particular are emerging as important actors in the agri-food system. The importance of China in the transition of the third food regime has been noted elsewhere (McMichael 2019; Belesky and Lawrence 2018), yet the growing involvement and power of Chinese tech companies has so far gone largely unnoticed.

In terms of *labor and production*, we found that the flexible and precarious conditions of labor typical of the third food regime might be deepened if robotics reduce the need for farm laborers and, as such, their bargaining power and political support (see also Carolan 2019). Our data also suggests that digital technologies might enable new forms of digital Taylorism on the fields and in greenhouses and food storage by increasing surveillance and new forms of work standardization, as well as farmers’ reliance on data-based advice. This might lead to a deskilling of (farm) laborers and a loss of agricultural and ecological knowledge.

In a context where the neoliberal paradigm of the third food regime is fragmenting, we are currently witnessing the start of a re-regulation of the agriculture and food sector, particularly in the EU, due to concerns over the climate crisis and food safety. Standards and regulations might become a further driver of digitalization: if farmers and food traders have to prove their compliance with complex national or EU policies and bi- and multilateral trade agreements, digital technologies might become a prerequisite to provide the necessary information.

Finally, we identified a new tendency for data to be used by agri-food companies to generate value and increase control over farmers. As Fraser (2019) states, land grabs are now accompanied by *data grabs*, when companies collect and use large amounts of previously proprietary, private or unused agricultural data through farm management platforms, digital marketplaces and digital supply chain technologies. More democratic alternatives such as open source platforms remain marginalized, not least because they lack capital to fully compete with proprietary systems.

Based on the digital technologies we analyzed in this article, we see digitalization as taking on a twofold role in the current food regime. On the one hand, we believe that digitalization is deepening some of the existing and still relatively stable characteristics of the third food regime. As a continuation of the importance of ICTs, which enabled global commodity chains in the first place, digitalization allows for even stronger control by the retail sector over food production and consumption (even as traditional supermarkets face growing competition from internet-based tech companies) and increased efficiency along the chain. On the other hand, we believe that digital technologies also add to the transitional tendencies of the regime. We understand the incorporation of digital technologies into the agri-food sector as part of a broader restructuring towards ‘digital capitalism,’ which Staab (2019, p. 43) argues is characterized by a system of proprietary markets and the commodification of data that is about to replace the neoliberal free market paradigm. We see agri-food companies increasingly attempting to establish such business models alongside their traditional strategies. This is evidenced in the fierce competition to attract users to farm management platforms and online marketplaces, and to establish a monopoly over the ‘market’ of farmers and the buyers of agricultural products. Agri-food companies have started to extract value from the data they collect and to use digital technologies to lock-in farmers into their own product ecosystems (e.g. through farm inputs or machinery), without facing effective government regulation regarding the protection of farmers’ data. Furthermore, digital tech companies are moving into the agri-food sector. At a stage of capitalist development where large tech companies like Amazon and Google are the most profitable and powerful corporate entities, and digital tech capital is becoming ever more important, it is only logical that these actors are also increasingly shaping the production and consumption of food. Finally, going ‘digital’ or ‘smart’ is becoming a hegemonic model of economic and social development, in the agri-food system and beyond (Srnicek 2017). This narrative, when combined with the ‘green’ imperative identified by Friedmann (2005), has led to the notion of ‘climate-smart agriculture.’ This logic has been internalized by state actors and multilateral institutions, who legitimize their financial and political support for digital developments along the food commodity chain by citing environmental, climate and food safety issues. This poses an important challenge to the concept of agro-ecology favored by peasant movements around the globe (Newell and Taylor 2017).

Finally, our research suggests that questions of data sovereignty versus data privatization might become a site of political tension in the transition of the third food regime due to the growing role of data throughout the food commodity chain. While this has not been our main focus, future research should pay close attention to the challenges and opportunities of the digitalization of the agri-food sector for peasant movements, as well as to the development of digital alternatives, such as software products developed specifically for agro-ecological farming and digital farming communities, as well as those designed around the concepts of open source access and data sovereignty.

## Electronic supplementary material

Below is the link to the electronic supplementary material.

Electronic supplementary material 1 (DOCX 217 kb)

## Data Availability

Data collection for this article was conducted by the three authors of this paper through the review of reports and online research on agri-food companies. An overview of the reports analyzed for this paper is included as supplementary material.

## Footnotes

1 Search words were ‘agriculture,’ ‘farming,’ ‘food processing,’ ‘food trade,’ ‘food transport,’ ‘food packaging,’ ‘food retail’ and ‘food consumption,’ which were combined with ‘digital.’ We also included the combinations ‘food’ AND ‘industry 4.0’ and ‘e-commerce’ AND ‘food.’ The selection of our search terms might have missed contributions that do not refer to digital technologies as such but to more specific aspects of digitalization, for example (big) data.

2 After our preliminary search, we were missing reports by NGOs and political institutions, so we searched specifically for reports by NGOs active in the agrarian sector. We looked at the websites of GRAIN, Oakland Institute, Friends of the Earth, the Transnational Institute, the European Federation of Food, Agriculture and Tourism Trade Unions (EFFAT), the International Union of Food and Tourism (IUF), ILO and FAO. We also noted that some of the reports had a regional focus. Furthermore, as our initial search did not cover all world regions, we conducted another search based on the terms ‘digital’ AND ‘food,’ and ‘digital’ AND ‘agriculture’ AND ‘Latin America,’ ‘Brazil,’ ‘Mexico,’ ‘USA,’ ‘Canada’ on Google, Google Scholar and SCOPUS.

3 These reports and articles, as well as the search terms used to identify them, were only used for identifying specific technologies and building our empirical database. A wider variety of peer-reviewed articles was used for theoretical and analytical purposes.
