# Supplementary material for: Digitalization and the third food regime
Source: Agric Human Values. 2020 Oct 13;38(3):641–55. doi: 10.1007/s10460-020-10161-2 (PMC7550770; doi:10.1007/s10460-020-10161-2)
Supplement: Supplementary file 1 — Electronic supplementary material 1 (DOCX 217 kb) [file 10460_2020_10161_MOESM1_ESM.docx]

**Supplementary material for the article “Digitalization and the Third Food Regime”**

**References of analyzed reports and papers**

AIOTI. 2017. Digital Innovation Hubs: democratising digital technologies in agriculture: Alliance for Internet of Things Innovation. <https://aioti.eu/wp-content/uploads/2017/11/AIOTI_WG06_ADIHS_final.pdf>. Accessed 07 June 2020.

Arents, I. n.D. Realising the potential of digitisation for food production, distribution and nutrition. Brussels: Flanders' Food.

Aulbur, W., R. Henske, W. Uffelmann, and G. Schelfi. 2019. Farming 4.0: How precision agriculture might save the world. Precision farming improves farmer livelihoods and ensures sustainable food production. Munich: Roland Berger.

Bakan, J., A. Gurumurthy, N. Chami, L. Jones, G. Adoración, A. Deneault, M. Vander Stichele, B. Brennan, G. Berrón, M. Kelly, T. Maso, N. Prins, B. Francis, and A. Eliseeva. 2020.The Corporation: State of Power 2020. Brussels: Transnational Institute.

Banker, R. D., S. Mitra, and V. Sambamurthy. 2011. The Effect of Digital Trading Platforms on Commodity Prices in Agricultural Supply Chains. *MIS Quarterly* 35(3): 599-611.

Brewer, S., and S. Pearson. 2019. Digital food. Briefing Document: digital technologies for improving productivity in food manufacturing: Internet of Food Things and Centre for Sustainable Manufacturing Recycling Technologies (SMART). <https://doi.org/10.5281/zenodo.3457891>. Accessed 08 June 2020.

Brice, J. 2018. Food in the Platform Economy: Understanding and Governing Emerging Digital Marketplaces. London: CARR.

Bundesverband der Verbraucherzentralen. 2017. The digitalisation of food. Berlin: Verbraucherzentrale Bundesverband.

Deichmann, U., A. Goyal, and D. Mishra. 2016. Will Digital Technologies Transform Agriculture in Developing Countries? World Bank Policy Research Working Paper 7669: World Bank Group. <http://documents.worldbank.org/curated/en/481581468194054206/pdf/WPS7669.pdf>. Accessed 10 June 2020.

Deloitte. 2018. Industry 4.0 in Food Industry. India Food Report: Deloitte. <https://www2.deloitte.com/content/dam/Deloitte/in/Documents/consumer-business/immersion/Deloitte-Chaper-india-Food-Report.pdf>. Accessed 12 June 2020.

Dryancour, Gi.. 2017. Smart Agriculture for All Farms. What needs to be done to help small farms access Precision Agriculture? How can the next CAP help? Brussels: European Agricutural Machinery Association (CEMA).

Eip-Agri. 2017. Agroinnovation Magazine: Where Research and Practice Meets. Vol 4. <https://op.europa.eu/en/publication-detail/-/publication/44cedd95-e46f-11e7-9749-01aa75ed71a1/language-en/format-PDF/source-61052725>. Accessed 13 June 2020.

Eip-Agri. 2018. Agroinnovation Magazine: Where Research and Practice Meets. Vol 5. <https://op.europa.eu/en/publication-detail/-/publication/01367860-028a-11e9-adde-01aa75ed71a1/language-en/format-PDF/source-83456312>. Accessed 13 June 2020.

Eip-Agri. 2019. Agroinnovation Magazine: Where Research and Practice Meets. Vol 6. <https://ec.europa.eu/eip/agriculture/sites/agri-eip/files/eip-agri_agrinnovation_magazine_6_2019_en_web.pdf>. Accessed 13 June 2020.

Euractiv. 2020. How is tech revolutionising the agricultural sector?. <https://en.euractiv.eu/wp-content/uploads/sites/2/special-report/EA-SPECIAL-REPORT-DGAGRI-EN-02032020.pdf>. Accessed 10 June 2020.

Garcia, K.. 2018. How some retailers are innovating in the digital grocery space. Digital transformation is permeating all aspects of selling food and beverage. eMarketer. <https://www.emarketer.com/content/how-some-retailers-are-innovating-in-the-digital-grocery-space>. Accessed 13 June 2020.

GE. n.D. Digital Transformation: A bold future for MENA food and beverage manufacturing: General Electric. <https://www.ge.com/>. Accessed 12 June 2020.

Geijer, T.. 2019. Food tech: technology in the food industry: ING Bank. <https://think.ing.com/reports/food-tech-technology-in-the-food-industry/>. Accessed 14 June 2020.

Global Information Society Watch. 2019. Artificial intelligence: Human rights, social justice and development. USA: Association for Progressive Communications (APC); Swedish International Development Cooperation Agency (Sida) and ARTICLE 19. <https://giswatch.org/2019-artificial-intelligence-human-rights-social-justice-and-development>.

GRAIN. 2016. Editorial. Supermarket Watch Asia bulletin 1. <https://us12.campaign-archive.com/?u=8496963c2e947c60c2bd03f02&id=7939244fc8&e=9ea630f8be>. Accessed 15 June 2020.

GRAIN. 2018. Top E-commerce companies move into retail. Supermarket Watch Asia bulletin 10. https://grain.org/en/article/5957. Accessed 15 June 2020.

GRAIN. 2018.The Belt and Road Initiative: Chinese Agribusiness going Global. Barcelona: GRAIN.

GRAIN. 2018. Summary report of the public seminar "Supermarkets: today’s food source - Trends and impact": GRAIN and BiothaiFoundation. https://www.grain.org/en/article/6051-summary-report-of-the-public-seminar-supermarkets-today-s-food-source-trends-and-impact. Accessed 15 June 2020.

GRAIN. 2019a. Black Diwali: E-commerce eats away at the livelihoods of small retailers. Supermarket watch Asia bulletin 16. <https://grain.org/en/article/6362>. Accessed 14 June 2020.

GRAIN. 2019b. Reflections from India and Thailand on the modern retail invasion. Supermarket watch Asia bulletin 15. <https://www.grain.org/en/article/6317-reflections-from-india-and-thailand-on-the-modern-retail-invasion>. Accessed 17 June 2020.

GRAIN. 2019c. Where’s the place for small farmers and traders in the digital marketing world?: GRAIN. Supermarket watch Asia bulletin 16. <https://grain.org/en/article/6231-where-s-the-place-for-small-farmers-and-traders-in-the-digital-marketing-world>. Accessed 14 June 2020.

Haman, K. 2017. New Professions and Career Paths in the food and drink industry: Delivering High-Level food industry skills in the digital economy. Brussels: EFFAT and FoodDrinkEurope.

Hasnan, N. Z., and Y. Yusoff. 2018. Short review: Application Areas of Industry 4.0 Technologies in Food Processing Sector. Paper presented at the IEEE Student Conference on Research and Development (SCOReD), Malaysia.

Jouanjean, M.-A. 2019. Digital Opportunities for Trade in the Agriculture and Food Sectors. *OECD Food, Agriculture and Fisheries Papers* 122: OECD. <http://dx.doi.org/10.1787/91c40e07-en>. Accessed 12 June 2020.

Lowe, B., I. Fraser, and D. M. Souza-Monteiro. 2015. A Change for the Better? Digital Health Technologies and Changing Food Consumption Behaviors. *Psychology & Marketing* 32 (5). <https://doi.org/10.1002/mar.20802>.

Malabo Montpellier Panel. 2019. Byte by Byte. Policy Innovation for Transforming Africa’s Food System with Digital Technologies. Dakar.

McKinsey & Company. 2020. Future of retail operations: Winning in a digital era: McKinsey & Company. https://www.mckinsey.com/industries/retail/our-insights/future-of-retail-operations-winning-in-a-digital-era. Accessed 14 June 2020.

McNamara, C.. n.D. Industry Briefing: Digitalization in Food & Beverage: Siemens. <https://www.plm.automation.siemens.com/media/global/it/DE4FB-Industry-Briefing-Digitalization-In-Food-And-Beverage-66023_tcm56-17780.pdf>. Accessed 18 June 2020.

Moller, B., A. Voglhuber-Slavinsky, E. Dönitz, and A. Rosa. 2019. 50 trends influencing Europe's food sector by 2035. Karlsruhe: Frauenhofer Institute for Systems and Innovations Research ISI.

Mooney, P. 2018. Blocking the chain. Industrial food chain concentration, Big Data platforms and food sovereignty solutions. Berlin: ETC Group, GLOCON, INKOTA, Rosa-Luxemburg Foundation.

Nestlé. 2019. Lagebericht. Veyvey: Nestlé.

PA Consulting. 2018. Transforming Agriculture with data driven insights. How to prosper in the evolving market for digital agritech. London.

Paunov, C., and S. Planes-Satorra. 2019. How are digital technologies changing innovation? Evidence from agriculture, the automotive industry and retail. *OECD Science, Technology and Industry Policy Papers* 74: OECD.

Phillips, P. W.B., J.-A. Relf-Eckstein, G. Jobe, and B. Wixted. 2019. Configuring the new digital landscape in western Canadian agriculture. *NJAS - Wageningen Journal of Life Sciences.* https://doi.org/10.1016/j.njas.2019.04.001.

Pigatto, G., J. Guilherme De Camargo Ferraz Machado, A. Dos Santos Negreti, and L. M. Machado. 2017. Have you chosen your request? Analysis of online food delivery companies in Brazil. *British Food Journal* 119 (3): 639-657.

Pinder, S., P. Walsh, M. Orndorff, E. Milton, and J. Trescott. 2017. The future of food. New realities for the industry: Accenture. <https://www.accenture.com/us-en/_acnmedia/pdf-70/accenture-future-of-food-new-realities-for-the-industry.pdf>. Accessed 17 June 2020.

Raheem, D., M. Shishaev, and V. Dikovitsky. 2019. Food System Digitalization as a Means to Promote Food and Nutrition Security in the Barents Region. *Agriculture 9*. <https://doi.org/10.3390/agriculture9080168>.

Rhodes, J.. 2018. Digital Transformation helps drive tighter cold chain integration: Emerson Commercial. <https://climate.emerson.com/documents/digital-transformation-helps-drive-tighter-cold-chain-integration-en-us-5007044.pdf>. Accessed 16 June 2020.

Saha, A. 2019. Role of Digital Technologies in Food Sustainability. Foods for Global Sustainability Network Newletter 1. [https://www.researchgate.net/publication/333798506. Accessed 17 June 2020](https://www.researchgate.net/publication/333798506.%20Accessed%2017%20June%202020). Accessed 17 June 2020.

Schneider, T., K. Eli, C. Dolan, and S. Ulijaszek. 2018. *Critical Food Studies: Digital Food Activism*. New York: Routledge.

Soma, K., M.-J. Bogaardt, K. Poppe, S. Wolfert, G. Beers, and D. Urdu. 2019. Research for AGRI Committee. Impacts of the digital economy on the food chain and the CAP. Brussels: European Parliament.

Song, D.-H. 2017. Behind Amazon's acquisition over Whole Foods: the next phase of food distribution. Supermarket Watch Asia 7: GRAIN. <https://www.grain.org/es/article/5782-behind-amazon-s-acquisition-over-whole-foods-the-next-phase-of-food-distribution>. Accessed 18.06.2020.

Spence, C., M. Mancini, and G. Huisman. 2019. Digital Commensality: Eating and Drinking in the Company of Technology*. Frontiers in Psychology* 10: 2252. doi:10.3389/fpsyg.2019.02252.

Townsend, R. J., D. Lampietti, K. Treguer, M. Schroeder, A. Juergenliemk, E. Hasiner, A. Horst, and A. Hakobyan. 2019. FUTURE of FOOD: Harnessing Digital Technologies to Improve Food System Outcomes. Washington, DC: World Bank Group.

Trendov, N. M., S. Varas, and M. Zeng. 2019. Digital Technologies in Agriculture and Rural Areas. Status Report. Rome: FAO.

Tsan, M., S. Totapally, M. Hailu, and B. K. Addom. 2019. The Digitalisation of African Agriculture Report. Wageningen: The Technical Centre for Agricultural and Rural Cooperation (CTA) and Dalberg Advisors.

Vanderroost, M., P. Ragaert, J. Verwaeren, B. De Meulenaer, B. De Baets, and F. Devlieghere. 2017. The digitization of a food package’s life cycle: Existing and emerging computer systems in the pre-logistics phase. *Computers in Industry* 87: 1-14. doi:10.1016/j.compind.2017.02.002.

Weinswig, D. 2016. The Digitalization of food: Grocery retail and food service. Hong Kong; London; New York: FUNG Global Retail & Technology.
